# Mapping poverty using mobile phone and satellite data

**DOI:** 10.1098/rsif.2016.0690

**Published:** 2017-02

**Authors:** Jessica E. Steele, Pål Roe Sundsøy, Carla Pezzulo, Victor A. Alegana, Tomas J. Bird, Joshua Blumenstock, Johannes Bjelland, Kenth Engø-Monsen, Yves-Alexandre de Montjoye, Asif M. Iqbal, Khandakar N. Hadiuzzaman, Xin Lu, Erik Wetter, Andrew J. Tatem, Linus Bengtsson

**Affiliations:** 1 Geography and Environment, University of Southampton, University Road, Building 44, Southampton, UK; 2 Flowminder Foundation, Roslagsgatan 17, Stockholm, Sweden; 3 Telenor Group Research, Oslo, Norway; 4 School of Information, University of California, Berkeley, CA, USA; 5 Data Science Institute, Imperial College London, London, UK; 6 Grameenphone Ltd, Dhaka, Bangladesh; 7 Public Health Sciences, Karolinska Institute, Stockholm, Sweden; 8 College of Information System and Management, National University of Defense Technology, Changsha, Hunan, People's Republic of China; 9 Stockholm School of Economics, Saltmätargatan 13-17, Stockholm, Sweden; 10 John E Fogarty International Center, National Institutes of Health, Bethesda, MD, USA

**Keywords:** poverty mapping, mobile phone data, Bayesian geostatistical modelling, remote sensing

## Abstract

Poverty is one of the most important determinants of adverse health outcomes globally, a major cause of societal instability and one of the largest causes of lost human potential. Traditional approaches to measuring and targeting poverty rely heavily on census data, which in most low- and middle-income countries (LMICs) are unavailable or out-of-date. Alternate measures are needed to complement and update estimates between censuses. This study demonstrates how public and private data sources that are commonly available for LMICs can be used to provide novel insight into the spatial distribution of poverty. We evaluate the relative value of modelling three traditional poverty measures using aggregate data from mobile operators and widely available geospatial data. Taken together, models combining these data sources provide the best predictive power (highest *r*^2^ = 0.78) and lowest error, but generally models employing mobile data only yield comparable results, offering the potential to measure poverty more frequently and at finer granularity. Stratifying models into urban and rural areas highlights the advantage of using mobile data in urban areas and different data in different contexts. The findings indicate the possibility to estimate and continually monitor poverty rates at high spatial resolution in countries with limited capacity to support traditional methods of data collection.

## 1. Background

In 2015, approximately 700 million people lived in extreme poverty [1]. Poverty is a major determinant of adverse health outcomes including child mortality [2], and contributes to population growth [3], societal instability and conflict [4]. Eradicating poverty in all its forms remains a major challenge and the first target of the Sustainable Development Goals (SDGs) [5]. To eradicate poverty, it is crucial that information is available on where affected people live. Such data improve the understanding of the causes of poverty, enable improved allocation of resources for poverty alleviation programmes, and are a critical component for monitoring poverty rates over time. The latter issue is especially pertinent for efforts aimed at reaching the SDGs, which need to be monitored at national and subnational levels over the coming 15 years [5].

The definition of poverty and the measurement methods used to identify poor persons are part of a longstanding discussion in development economics [6–9]. Different approaches exist to calculate indicators of living standards, including the construction of unidimensional and multidimensional indices, as well as the use of monetary or non-monetary metrics. A further discussion for living standard indices regards the methods used to set appropriate thresholds (poverty lines) under which a person is defined as poor [10–12]. Monetary-based metrics identify poverty as a shortfall in consumption (or income) and measure whether households or individuals fall above or below a defined poverty line [13,14]. By contrast, asset-based indicators define household welfare based on asset ownership (e.g. refrigerator, radio or bicycle), dwelling characteristics, and access to basic services like clean water and electricity [15]. Moreover, poverty indicators can capture the status of a household or individual at a given point in time, or identify chronic versus transient poverty over time [14,16–18].

Every approach used to calculate indicators of living standards for a population has its advantages and disadvantages, and each indicator discerns different characteristics of the population. Consumption data can be highly noisy due to recall error or because expenditures occurred outside the period captured in surveys, but provide a better shorter-term concept of poverty [19,20]. Asset-based measures have been regarded as a better proxy for the long-term status of households as they are thought to be more representative of permanent income or long-term control of resources [20–22]. The same population can be ranked quite differently along a poverty distribution when comparing consumption and asset-based measures and many assumptions are necessarily accepted in order do such comparisons. These include assumptions that the data represent the same populations in the same time period; that the indicators are well matched in their wording and response options; and that the poverty measures have a similar distribution of responses [20,23]. Furthermore, it is difficult to compare asset-based measures to income or consumption as it is not straightforward to link the productive potential of a household to their assets owned; this can be particularly relevant in rural areas where the return on physical assets can be strongly environmentally related and interactions among assets may be important [24]. These factors necessitate a flexible approach to modelling poverty as indicators representing asset-based, consumption-based and income-based measures are not necessarily expected to produce similar results.

While numerous high-resolution indicators of human welfare are routinely collected for populations in high-income countries, the geographical distribution of poverty in low- and middle-income countries (LMICs) is often uncertain [25]. Small area estimation (SAE) forms the standard approach to produce sub-national estimates of the proportion of households in poverty. SAE uses statistical techniques to estimate parameters for sub-populations by combining household survey and census data to use the detail in household surveys and the coverage of the census. Common variables between the two are used to predict a poverty metric across the population [26–28]. These techniques rely on the availability of census data, which are typically collected every 10 years and often released with a delay of one or more years, making the updating of poverty estimates challenging. Recently, there are promising signs that novel sources of high-resolution data can provide an accurate and up-to-date indication of living conditions. In particular, recent work illustrates the potential of features derived from remote sensing and geographic information system data [29–35] (hereafter called RS data) and mobile operator call detail records (CDRs) [36–39]. However, the predictive power in integrating these two data sources, and their ability to estimate different measures of poverty has not been evaluated.

RS and CDR data capture distinct and complimentary correlates of human living conditions and behaviour. For example, RS data of physical properties, such as rainfall, temperature and vegetation capture information related to agricultural productivity, while distance to roads and cities reflects access to markets and information. Similarly, monthly credit consumption on mobile phones and the proportion of people in an area using mobile phones indicate household access to financial resources, while movements of mobile phones and the structure and geographical reach of the calling networks of individuals may be correlated with remittance flows and economic opportunities [39–41].

RS and CDR data are generated at different spatial scales, which further complement each other. The CDR indicators used in this study are derived from data aggregated at the level of the physical cell towers to preserve the privacy of individual subscribers. Thus, the spatial resolution of these data is determined by tower coverage, which is larger in rural areas and fine-scaled in urban areas. By contrast, RS data can be relatively coarse in urban areas and only capture physical properties of the land. As RS and CDR data are continually collected, the ability to produce accurate maps using these data types offers the promise of ongoing subnational monitoring required by the SDGs.

Here, we use overlapping sources of RS, CDR and traditional survey-based data from Bangladesh to provide the first systematic evaluation of the extent to which different sources of input data can accurately estimate three different measures of poverty. To date, the predictive power in integrating these data sources, and their ability to estimate different measures of poverty, has not been evaluated. We use hierarchical Bayesian geostatistical models (BGMs) to construct highly granular maps of poverty for three commonly used indicators of living standards: the Demographic and Health Surveys (DHS) Wealth Index (WI); an indicator of household expenditures (Progress out of Poverty Index, PPI) [42] and reported household monetary income. We additionally compare our results with previous poverty estimates for Bangladesh at coarser and finer resolutions.

## 2. Material and methods

### 2.1. Spatial scale and data processing

**Figure 1.**
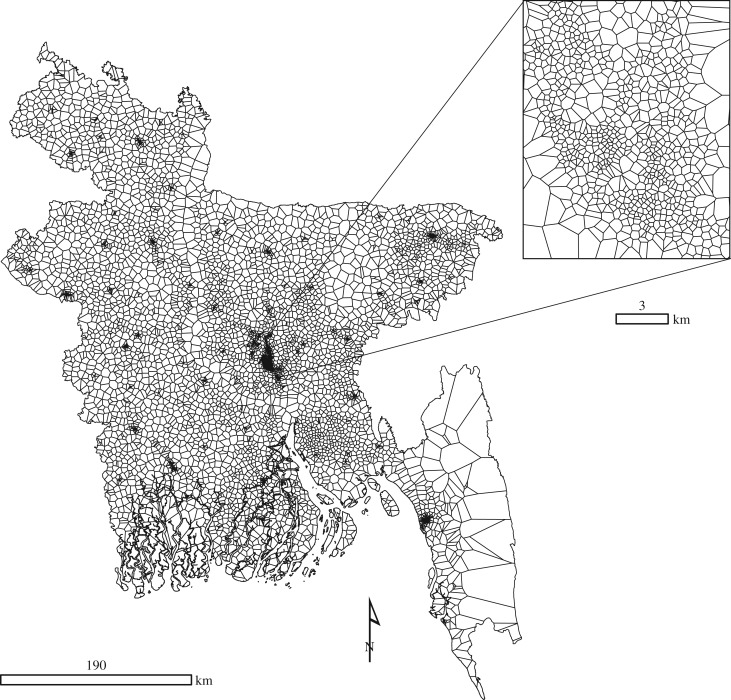
Spatial structure of Voronoi polygons based on the configuration of mobile phone towers in Bangladesh. The zoom window shows the spatial detail of Dhaka.

All data used in this study were processed to ensure that projections, resolutions and extents matched. The spatial scale of analysis was based on approximating the mobile tower coverage areas using Voronoi tessellation [43] and models were built on the scale of the Voronoi polygons (figure 1). This allowed us to maintain the fine spatial detail in mobile phone data within urban areas, as Voronoi polygon size, and corresponding spatial detail, varies greatly from urban to rural areas (minimum 60 m, maximum 5 km) as shown in the figure. All datasets were then summarized to spatially align with these polygons. In practice, each polygon was assigned RS and CDR values representing the mean, sum or mode of the corresponding data. The survey data are matched to the Voronois based on the GPS located lat/long of PPI data, the lat/long representing the centroid of each DHS cluster, and the home tower of each income survey respondent. Where multiple points from the same output (WI, PPI and income) fell within the same polygon, we used the mean aggregated value.

### 2.2. Poverty data

We used three geographically referenced datasets representing asset, consumption and income-based measures of wellbeing in Bangladesh (see the electronic supplementary material, figure S1 and section A.1). These data were obtained from three sources: the 2011 Bangladesh DHS, the 2014 FII survey [44] with data collected on the PPI (www.progressoutofpoverty.org) and national household surveys conducted by Telenor Group subsidiary Grameenphone (GP) between November 2013 and March 2014 collecting household income data.

The DHS WI is constructed by taking the first principal component of a basket of household assets and housing characteristics such as floor type and ceiling material, which explains the largest percentage of the total variance, adjusting for differences in urban and rural strata [45]. A final composite combined score is then used as a WI whereby each household is assigned its correspondent quintile in the distribution and each individual belonging to the same household shares the same WI score. A higher score implies higher socioeconomic status (range = −1.45 to 3.5). Here, we used aggregated average WI scores per primary sampling unit (PSU) for 600 PSUs (207 in urban areas and 393 in rural areas) to estimate the mean WI of sampled populations residing in each Voronoi polygon.

The PPI is a measurement tool built from the answers to 10 questions about a household's characteristics and asset ownership, scored to compute the likelihood the household is living above or below a poverty line. In Bangladesh, these poverty scorecard questions were determined using data from the 2010 Household Income and Expenditure Survey (HIES) [42,46], and used in a nationally representative survey of 6000 Bangladeshi adults undertaken in 2014 [44]. Together with basic demographics and access to financial services information, the 10 questions needed to construct the PPI were collected. These data were used to assign a poverty measure to each individual interviewed: the likelihood they have *per capita* expenditure above or below a poverty line. Here, we estimate the mean likelihood (range = 12.3–99.7%) of populations residing in each Voronoi polygon to be below the $2.50 a day poverty line.

Income data were obtained from two independent, sequential household surveys run by GP. For each survey, face-to-face interviews were conducted with 90 000 individuals, and their corresponding household income was collected, together with basic demographic information for each survey participant (e.g. gender, age, profession, education) and phone usage. Respondents were directly asked about income and were requested to place themselves within pre-set income bins. Among GP subscribers, CDRs were successfully linked to phone numbers for 76 000 participants. Here we converted income bins to USD (range = 0–1285$) and modelled the average USD for each Voronoi polygon.

### 2.3. CDR and RS data

CDR features were generated from four months of mobile phone metadata collected between November 2013 and March 2014. GP subscribers consented to the use of their data for the analysis. GP, the largest mobile network operator in Bangladesh, had 48 million customers at the time of the analysis, with a network covering 99% of the population and 90% of the land area [47]. CDR features range from metrics such as basic phone usage, top-up patterns, and social network to metrics of user mobility and handset usage. These features are easily made available in data warehouses and do not rely on complex algorithms. They include various parameters of the corresponding distributions such as weekly or monthly median, mean and variance (see the electronic supplementary material).

We further identified, assembled and processed 25 raster and vector datasets into a set of RS covariates for the whole of Bangladesh at a 1 km spatial resolution. These data were obtained from existing sources and produced *ad hoc* for this study to include environmental and physical metrics likely to be associated with human welfare [31,33,48–50] such as vegetation indices, night-time lights, climatic conditions, and distance to roads or major urban areas. A full summary of assembled covariates is provided in the electronic supplementary material.

### 2.4. Covariate selection

Prior to statistical analyses, all CDR and RS covariate data were log transformed for normality. Bivariate Pearson's correlations were computed for each pair of covariates to assess multicollinearity, and for high correlations (*r* > 0.70), we eliminated covariates that were less generalizable outside Bangladesh. For example, population data are widely available (e.g. www.worldpop.org.uk/) but births data may not be; similarly, volumes of calls could be computed and compared across countries, but charges may be country-specific.

To identify the set of predictors most suitable for modelling the WI, PPI, and income data, we employed a model selection stage as is common in statistical modelling [51]. For this we used non-spatial generalized linear models (glms), implemented via the R *glmulti* package [52,53], to build every possible non-redundant model for every combination of covariates. Models were built on a randomly selected 80% of the data to guard against overfitting. Models were chosen using Akaike's information criterion (AIC), which ranks models based on goodness of fit and complexity, while penalizing deviance [52]. A full IC-based approach such as this allows for multi-model inference. Where multiple glms had near-identical AIC values, we selected the model with the fewest number of covariates. For the CDR data only, we used forward and backward stepwise selection (*p* = 0.05) prior to model selection to reduce the initial CDR inputs from 150 to 30 or less. The covariate selection process was completed for all three poverty measures for national, urban and rural strata, and using RS-only, CDR-only and CDR–RS datasets (27 resulting models). This allowed us to explore differences in factors related to urban and rural poverty, as well as to explicitly compare the ability of RS-only, CDR-only and CDR–RS datasets to predict poverty measures. The resulting models were then used in the hierarchical Bayesian geostatistical approach (see the electronic supplementary material, tables S2*a–c*).

### 2.5. Prediction mapping

Using the models selected by the previous step, we employed hierarchical Bayesian geostatistical models (BGMs) to predict the three poverty metrics at unsampled locations across the population. We chose BGMs as they offer several advantages for addressing the limitations and constraints associated with modelling geolocated survey data. These include straightforwardly imputing missing data, allowing for the specification of prior distributions in model parameters and spatial covariance, and estimating uncertainty in the predictions as a distribution around each estimate [54,55].

Additionally, we needed to account for spatial autocorrelation in the data as they are aligned to the tower locations, which are clustered across varying spatial scales (described in §2.1 and figure 1). BGMs can achieve this through incorporating a spatially varying random effect. Here, the Voronoi polygons themselves form the neighbourhood structure for this spatial random effect, and neighbours are defined within a scaled precision matrix [56]. The matrix represents the spatially explicit processes that may affect poverty estimates. It is passed through a graph function in the model which assumes the neighbour relations are connected [57], that is all adjacent polygons share a boundary. This function accounts for the spatial covariance in the data by allowing observations to have decreasing effects on predictions that are further away.

**Figure 2.**
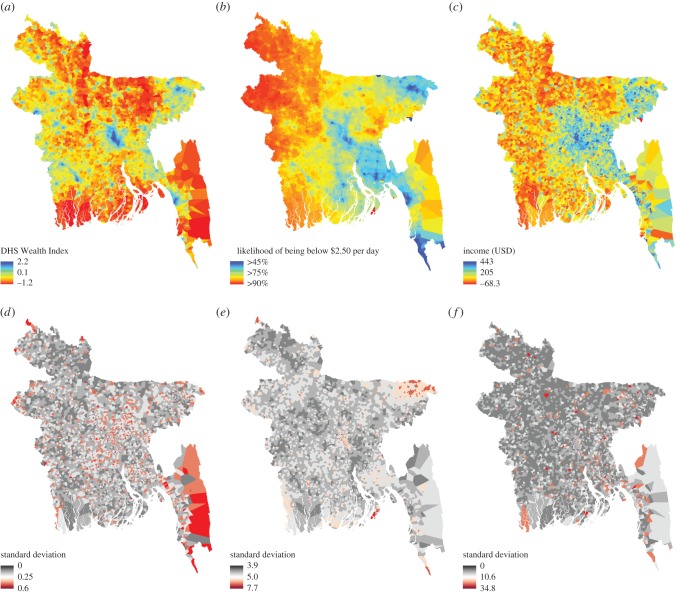
National level prediction maps for mean WI (*a*) with uncertainty (*d*); mean probability of households being below $2.50/day (*b*) with uncertainty (*e*); and mean USD income (*c*) with uncertainty (*f*). Maps were generated using call detail record features, remote sensing data and Bayesian geostatistical models. The maps show the posterior mean and standard deviation from CDR–RS models for the WI and income data (*a,c*), and the RS model for the PPI (*b*). Red indicates poorer areas in prediction maps, and higher error in uncertainty maps.

Here, all BGMs were implemented using integrated nested Laplace approximations (INLA) [58], which uses an approximation for inference and avoids the computational demands, convergence issues and mixing problems sometimes encountered by MCMC algorithms [59]. The model is fit using R-INLA, with the Besag model for spatial effects specified inside the function [60,61]. In the Besag model, Gaussian Markov random fields (GMRFs) are used as priors to model spatial dependency structures and unobserved effects. GMRFs penalize local deviation from a constant level based on the precision parameter *τ*, where the hyperpriors are loggamma distributed [56]. The hyperprior distribution governs the smoothness of the field used to estimate spatial autocorrelation [56]. The spatial random vector **x** = (*x*_1_, … ,*x_n_*) is thus defined as
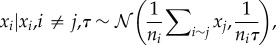
where *n_i_* is the number of neighbours of node *i*, *i ∼ j* indicates that the two nodes *i* and *j* are neighbours. The precision parameter *τ* is represented as
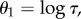
where the prior is defined on *θ*_1_ [60]. The geostatistical models defined for the WI, PPI and income data were applied to produce predictions of the each poverty metric for each Voronoi polygon as a posterior distribution with complete modelled uncertainty around estimates. The posterior mean and standard deviation for each polygon were then used to generate prediction maps with associated uncertainty (figure 2 and electronic supplementary material, figures S2–S6). Model performance was based on out-of-sample validation statistics calculated on a 20% test subset of data. Pearson product-moment correlation coefficient (*r*) (or Spearman's rho (*ρ*) for *n* < 100), root-mean-square-error (RMSE), mean absolute error (MAE) and the coefficient of determination (*r*^2^) were calculated for all BGMs. Finally, because glms do not incorporate prior information for model parameters, we ran each model through INLA while excluding the random spatial effect to obtain non-spatial Bayesian estimates and compare model fit and performance due to the explicit spatial process.

## 3. Results

We find models employing a combination of CDR and RS data generally provide an advantage over models based on either data source alone. However, RS-only and some CDR-only models performed nearly as well (table 1). While the combined CDR–RS model performed well in both urban (*r*^2^ = 0.78) and rural (*r*^2^ = 0.66) areas, and at the national level (*r*^2^ = 0.76), the performance of RS-only and CDR-only models was more context-dependent. For example, PPI and income models did not improve predictions in urban areas, but in rural areas the RS-only models performed nearly as well for both indicators. The fine spatial granularity of the resultant poverty estimates can be shown in figure 2, which shows the predicted distribution of poverty for all three measures. Spatially, the models exhibit higher uncertainty where fewer data are available, such as the peninsular areas surrounding Chittagong in the southeast where mobile towers are sparse. We also find that explicitly modelling the spatial covariance in the data was critically important. This resulted in improved predictions, lower error and better measures of fit based on cross-validation and the deviance information criteria (DIC), a hierarchical modelling generalization of the AIC [62] (electronic supplementary material, tables S3 and S4).

**Table 1.** Cross-validation statistics based on a random 20% test subset of data for all Bayesian geostatistical models.

poverty metric	model	*r*^2^	RMSE
whole country			
DHS WI	CDR–RS	0.76	0.394
	CDR	0.64	0.483
	RS	0.74	0.413
PPI	CDR–RS	0.25	57.907
	CDR	0.23	58.562
	RS	0.32	57.439
income	CDR–RS	0.27	105.465
	CDR	0.24	107.155
	RS	0.22	108.682
urban			
DHS WI	CDR–RS	0.78	0.424
	CDR	0.70	0.552
	RS	0.71	0.433
PPI	CDR–RS	0.00	60.128
	CDR	0.03	60.935
	RS	0.00	60.384
income	CDR–RS	0.15	168.452
	CDR	0.15	172.738
	RS	0.05	176.705
rural			
DHS WI	CDR–RS	0.66	0.402
	CDR	0.50	0.483
	RS	0.62	0.427
PPI	CDR–RS	0.18	57.397
	CDR	0.17	57.991
	RS	0.21	57.162
income	CDR–RS	0.14	81.979
	CDR	0.13	82.773
	RS	0.23	76.527

Separating estimation by urban and rural regions further highlights the importance of different data in different contexts (electronic supplementary material, tables S2*a–c*). Night-time lights, transport time to the closest urban settlement, and elevation were important nationally and in rural models; climate variables were also important in rural areas. Distances to roads and waterways were significant in urban and rural strata. In general, the addition of CDR data did not change the selection of RS covariates at any level. Top-up features derived from recharge amounts and tower averages were significant in every model, affirming their importance in poverty work. People predicted to be poorer top-up their phones more frequently in small amounts. Per cent nocturnal calls, and count and duration of SMS traffic were significant nationally. Mobility and social network features were important in all three strata. In urban areas, SMS traffic was important, whereas multimedia messaging and video attributes were key in rural areas.

**Figure 3.**
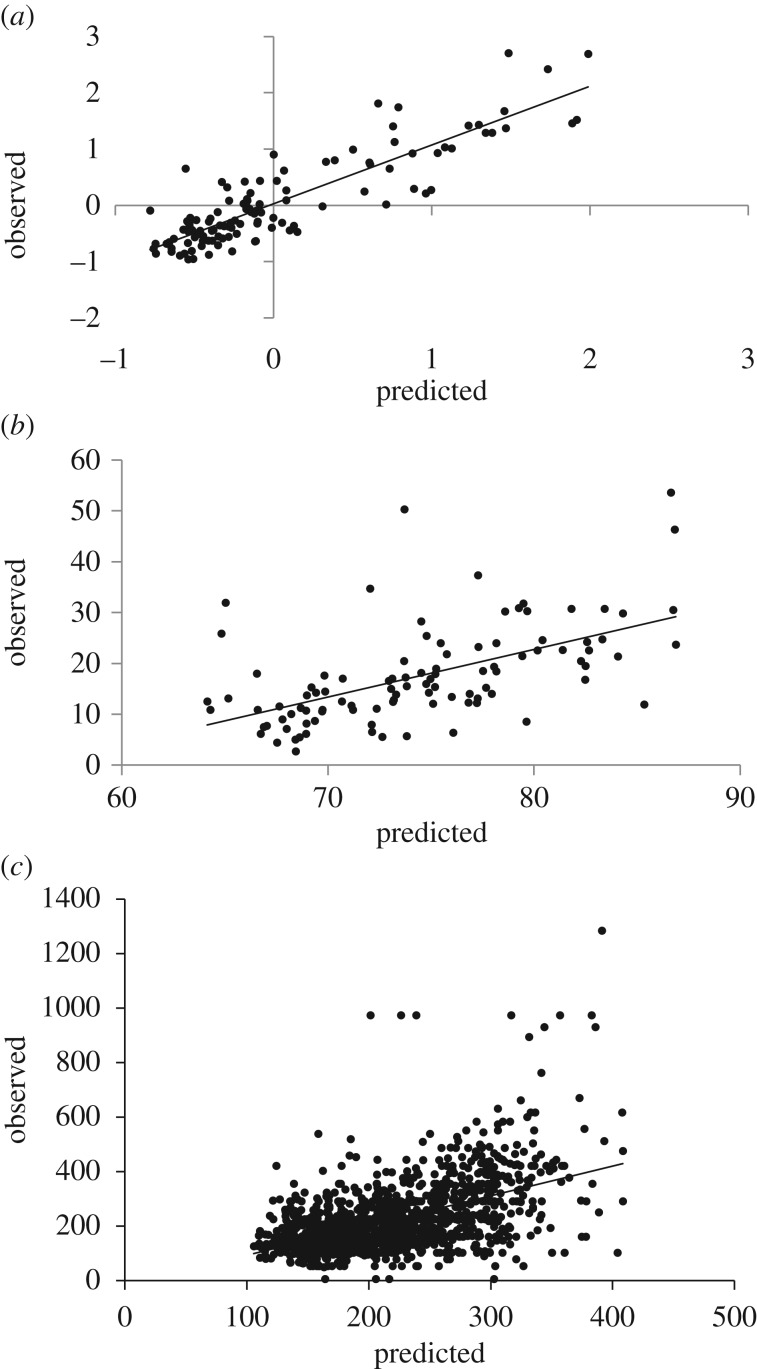
Out-of-sample observed versus predicted values for (*a*) DHS WI using mobile phone and remote sensing data: *r*^2^ = 0.76, *n* = 117, *p* < 0.001, RMSE = 0.394; (*b*) progress out of Poverty Index using remote sensing data: *r*^2^ = 0.32, *n* = 100, *p* < 0.001, RMSE = 57.439; and (*c*) income using mobile phone and remote sensing data: *r*^2^ = 0.27, *n* = 1384, *p* < 0.001, RMSE = 105.465.

Models were most successful at reconstructing the WI to model poverty (*r*^2^ = 0.76); consumption-based and income-based poverty proved more elusive. WI models have better fit, lower error and higher explained variance based on out-of-sample validation (figure 3). Combined CDR–RS data produced the best WI models and lowest error (*r*^2 (CDR–RS)^ = 0.76, *r*^2 (RS)^ = 0.74, *r*^2 (CDR)^ = 0.64; RMSE ^(CDR–RS)^ = 0.394, RMSE^(RS)^ = 0.413, RMSE^(CDR)^ = 0.483). However, for the PPI models, the best model predicting the probability of falling below $2.50/day was the RS-only model (figure 2*b,e*, *r*^2 (RS)^ = 0.32; RMSE^(RS)^ = 57.439). The model discerns many urban areas but also predicts areas with very low poverty likelihood and high uncertainty outside urban areas, especially around Sylhet in the northeast. Income predictions (figure 2*c,f*) show greater variation across the country, and the best national model was for combined CDR–RS data (*r*^2 (CDR–RS)^ = 0.27, RMSE ^(CDR–RS)^ = 105.465).

**Figure 4.**
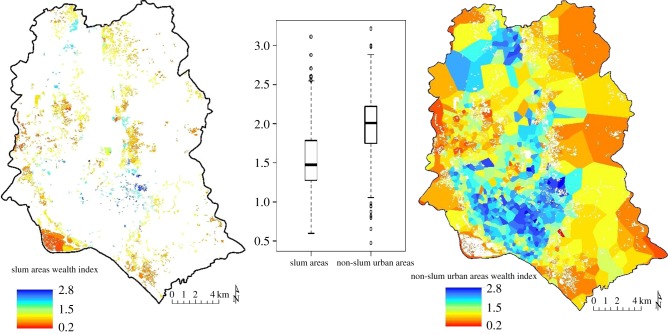
Comparison of predicted mean DHS WI values between slum and non-slum areas in Dhaka as delineated by Gruebner *et al*. [63] *t*_615_ =−17.2, *p* < 0.001. The 95% confidence interval using Student's *t*-distribution with 615 degrees of freedom is (−0.48, −0.38).

The resulting predictions line up well with existing SAE estimates for Bangladesh, and with high-resolution maps of slum areas in Dhaka. The urban CDR–RS model has the highest explained variance for any model (*r*^2 (CDR–RS_urb)^ = 0.78) and the urban CDR-only model outperforms the national CDR-only model (*r*^2 (CDR_urb)^ = 0.70). Precision and accuracy are slightly lower, but the improved correlation highlights the advantage of using CDRs within a diverse urban population. To explore this further, we compared our WI predictions against a spatially explicit dataset of slum areas in Dhaka [63] (figure 4). We find the mean predicted WI of slum and non-slum areas to be significantly different, *t*_615_ = −17.2, *p* < 0.001, predicting slum areas to be poorer than non-slum areas.

**Figure 5.**
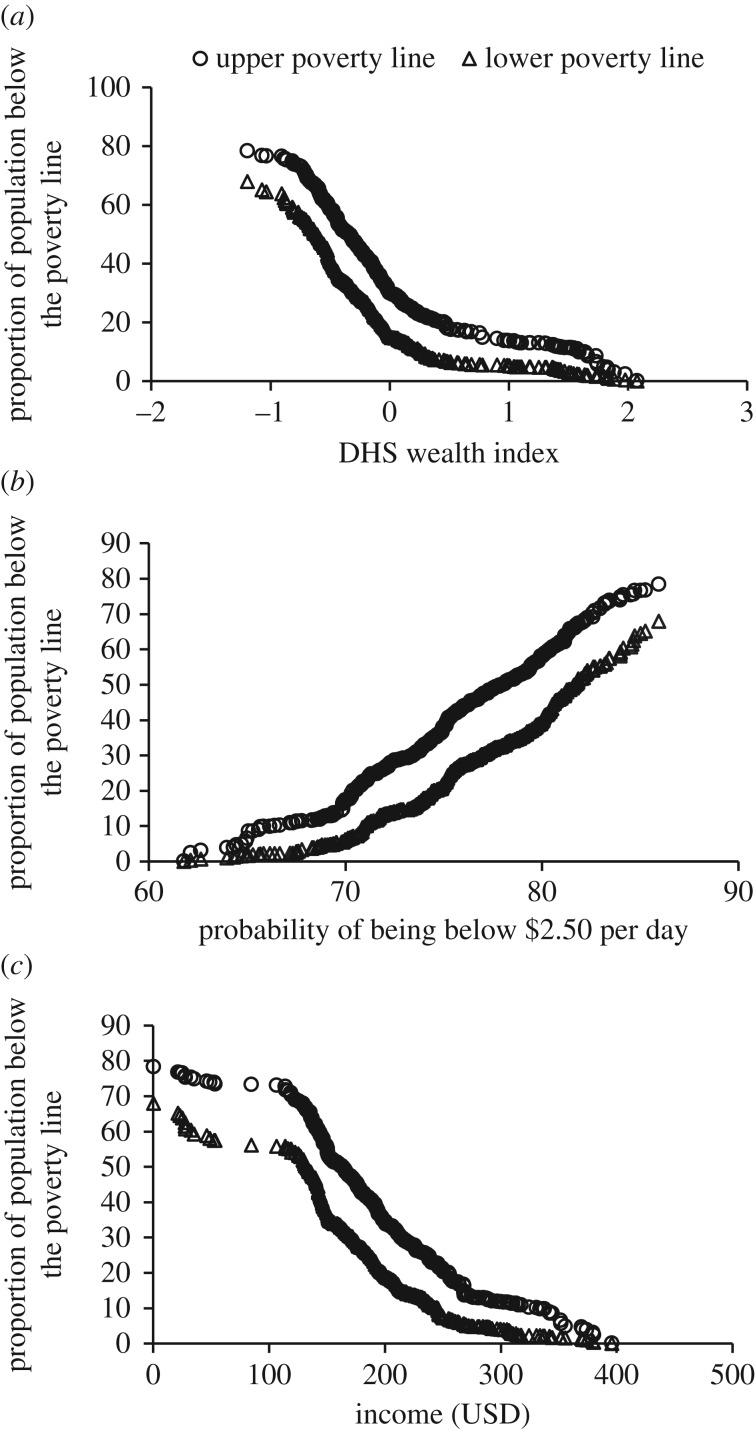
Comparison of the proportion of people falling below upper (circles) and lower (triangles) poverty lines estimated by Ahmad *et al*. [64] and (*a*) predicted mean WI using mobile phone and remote sensing data, (*b*) predicted probability of being below $2.50 per day using remote sensing data and (*c*) predicted income using mobile phone and remote sensing data. All models were predicted at the upazila scale (Admin unit 3). Pearson's *r* correlations: −0.91 and −0.86 for the WI; 0.99 and 0.97 for the PPI; and −0.96 and −0.94 for income, respectively (*p* < 0.001 for all models).

To compare our method to previous poverty estimates at administrative level 3 (upazila), we used the same methodology at the lower spatial resolution, using the upazila boundaries to form the random spatial effect in the model, and covariates from the best national level model for each poverty measure. We find strong correlations (*r* = −0.91 and −0.86 for the WI; 0.99 and 0.97 for the PPI; and −0.96 and −0.94 for income, respectively, *p* < 0.001 for all models) between our upazila predictions and earlier estimates of poverty derived from SAE techniques based on data from the 2010 Household Income and Expenditure (HIES) survey and 2011 census [64] (figure 5). The *r*-values reported for WI and income are negative at administrative level 3 because as the proportion of people below the poverty line as estimated by Ahmed *et al*. decreases, the WI value and income in USD of the sampled population increases. That is, people who are wealthier as estimated by the WI and income data are also less likely to live below the poverty line according to earlier estimates. The geostatistical method presented here thus accurately maps heterogeneities at small spatial scales while correlating well with earlier coarser estimates. All remaining WI, PPI and income prediction maps are provided in the electronic supplementary material.

## 4. Discussion

This work represents the first attempt to build predictive maps of poverty using a combination of CDR and RS data. The results demonstrate that CDR-only and RS-only models perform comparably in their ability to map poverty indicators, and that integrating these data sources provides improvement in predictive power and lower error. These results are promising as the CDR data here produce accurate, high-resolution estimates in urban areas not possible using RS data alone. As such, CDRs potentially allow for estimation of wealth at much finer granularity—including the neighbourhood or even the household or individual—than the current generation of RS technologies [36]. While CDRs are proprietary data, they are increasingly used in research, and have formed the basis for hundreds of published articles over the past few years [65]. They also provide significant advantages in temporal granularity: CDRs update in real-time versus RS data, which update far less frequently. Although in this study we have not used dynamic validation data, it is a clear future application for CDRs in real-time to better comprehend the dynamic nature of poverty.

The higher accuracy of predictions for the asset-based WI over other poverty metrics is presumably due to several factors. The predictive power for assets has been shown to be higher than for consumption [35] in addition to the aforementioned issues of survey question wording and response options [20,23]. Further, income and consumption can vary hugely by day, week, and can be related to changes in household size, job loss or gain, piecework or harvest outcomes. Assets and housing characteristics are generally considered more stable [20–22]. For the datasets used in this study, WI data are based on clusters of households, and this sampling strategy provides more robust estimates and less variability than the individually based PPI and income data. Greater success in predicting the WI is also presumably due to the WI measuring a wider range of living standard across the population. That is, the full range of distribution from poorest to wealthiest in the population is represented in these data. Alternatively, by considering a streamlined 10 questions, the PPI is meant to identify the poorest individuals in a population. Similarly, in the income data, there were very few respondents in higher income categories.

The higher error associated with CDR-only models is not surprising considering the noise inherent in these data. CDR features are derived from daily and weekly measurements aggregated over short temporal intervals, while RS covariates are generally comprised of long-term averages or comparatively less dynamic measures of location and access such as roads or proximity to urban centres. Bearing this in mind, we find CDR data useful for estimating poverty in the absence of ancillary datasets.

Our findings provide further support for correlations between socio-economic measures and night-time light intensity [36,48,49], access to roads and cities [50,66], entropy of contacts [37,40] and mobility features [39]. The universal coverage of cell towers across Bangladesh made it possible to predict poverty at high-resolution in both urban and rural areas. Within urban areas, the high correlation with maps of slums in Dhaka suggests we are capturing the poorest populations. Even if the poorest populations are not generating call data [36], and thus not included in the CDRs, we still see a clear difference in WI predictions between slum and non-slum areas using tower level CDR aggregates. This finding extends recent work which predicted wealth and poverty at the district level, but were unable to verify predictions at finer scales [36].

Using CDRs and RS data within BGMs to produce accurate, high-resolution poverty maps in LMICs offers a way to complement census-based methods and provide more regular updates. Regularly updated poverty estimates are necessary to enable subnational monitoring of the SDGs during intercensal years and are critical to ensure mobilization of resources to end poverty in all its dimensions as set out in SDG 1. Poverty estimates are time sensitive and become obsolete when factors such as migration rates, infrastructure, and market integration change [67]. Furthermore, the methods presented here offer a workaround to estimating poverty with household survey data, which can be time consuming and expensive to obtain.

To end poverty in all its dimensions, it is likely that methods that exploit information from, and correlations between, many different data sources will provide the greatest benefit in understanding the distribution of human living conditions. To leverage data from differing sample sizes, temporal and spatial scales, BGMs provide such a rigorous framework. This study further provides an example of how aggregated CDR data can be processed in such a way that detailed maps can be created without revealing sensitive user and commercial information. As insights from CDRs and other remote sensing data become more widely available, analysing these data at regular intervals could allow for dynamic poverty mapping and provide the means for operationally monitoring poverty. The combination of spatial detail and frequent, repeated measurements may distinguish the transitorily poor from the chronically poor, and allow for monitoring economic shocks [68]. This offers the potential for a fuller characterization of the spatial distribution of poverty and provides the foundation for evidence-based strategies to eradicate poverty. Researchers would do well to use the additional information and granularity afforded by CDR data with matched individual-based consumption data to further infer novel and useful information from mobile data.

## Supplementary Material

Supplementary Information (SI)
