# Supplementary material for: Mapping poverty using mobile phone and satellite data
Source: J R Soc Interface. 2017 Feb;14(127):20160690. doi: 10.1098/rsif.2016.0690 (PMC5332562; doi:10.1098/rsif.2016.0690)
Supplement: Supplementary Information (SI) [file rsif20160690supp1.pdf]

Mapping poverty using mobile phone and satellite data

J.E. Steele, P. Sundsoy, C. Pezzulo, V. Alegana, T. Bird, J. Blumenstock, J. Bjelland, K. Engo-Monsen, YA de Montjoye, A. Iqbal, K. Hadiuzzaman, X. Lu, E. Wetter, A.J. Tatem, and L. Bengtsson

Supplementary Information (SI)

Table of Contents

A. Input data 3

A.1. Geolocated survey data 3

A.1.1. Demographic and Health Survey Wealth Index 3

A.1.2. Progress out of Poverty Index 4

A.1.3. Market research household surveys and income data 4

A.2. Mobile phone call detail records (CDRs) 5

A.3. Remote Sensing-GIS covariates 6

A.3.1 GPS data displacement 6

B. Statistical analyses and prediction mapping 7

B.1. Covariate selection via generalized linear models 7

B.2. Prediction mapping via Bayesian geostatistical models 7

References 8

SI Tables and Figures 11

Table S1. Summary information for remote sensing-GIS and mobile phone call detail record datasets used for covariate selection and Bayesian geostatistical poverty mapping. 11

Table S2*A*. Wealth Index models for RS-only, CDR-only, and CDR+RS data: national, urban, and rural strata. 13

Table S2*B*. Progress out of Poverty Index models for RS-only, CDR-only, and CDR+RS data: national, urban, and rural strata. 14

Table S2*C*. Income models for RS-only, CDR-only, and CDR+RS data: national, urban, and rural strata. 15

Table S3. Comparison of r^2 and RMSE for INLA models run with only a structured spatial random effect (Spatial interpolation) and the full model (Spatial model + covariates). 16

Table S4. Comparison of deviance information criterion (DIC) model fit for Bayesian geostatistical models run with a structured spatial random effect (Spatial model) and without (Non-spatial model). 17

Figure S1. Survey sample locations for DHS wealth index (A), Progress out of Poverty Index (B), and income survey respondents (C). 18

Figure S2. National level prediction maps for mean wealth index (A) with uncertainty (D); mean probability of households being below $2.50/day (B) with uncertainty (E); and mean USD income (C) with uncertainty (F). Maps were generated using call detail record features only and Bayesian geostatistical models. Red indicates poorer areas in prediction maps, and higher error in uncertainty maps 19

Figure S3. National level prediction maps for mean wealth index (A) with uncertainty (D); mean probability of households being below $2.50/day (B) with uncertainty (E); and mean USD income (C) with uncertainty (F). Wealth index and income maps were generated using remote sensing data only; PPI maps were generated using call detail record features and remote sensing data. All maps were generated using Bayesian geostatistical models. Red indicates poorer areas in prediction maps, and higher error in uncertainty maps. 20

Figure S4. Stratified urban/rural prediction maps for mean wealth index (A) with uncertainty (D); mean probability of households being below $2.50/day (B) with uncertainty (E); and mean USD income (C) with uncertainty (F). Maps were generated using call detail record features only and Bayesian geostatistical models. Red indicates poorer areas in prediction maps, and higher error in uncertainty maps. Figure S5. Stratified urban/rural prediction maps for mean wealth index (A) with uncertainty (D); mean probability of households being below $2.50/day (B) with uncertainty (E); and mean USD income (C) with uncertainty (F). Maps were generated using call detail record features, remote sensing data, and Bayesian geostatistical models. Red indicates poorer areas in prediction maps, and higher error in uncertainty maps. 21

Figure S5. Stratified urban/rural prediction maps for mean wealth index (A) with uncertainty (D); mean probability of households being below $2.50/day (B) with uncertainty (E); and mean USD income (C) with uncertainty (F). Maps were generated using call detail record features, remote sensing data, and Bayesian geostatistical models. Red indicates poorer areas in prediction maps, and higher error in uncertainty maps. 22

Figure S6. Stratified urban/rural prediction maps for mean wealth index (A) with uncertainty (D); mean probability of households being below $2.50/day (B) with uncertainty (E); and mean USD income (C) with uncertainty (F). Maps were generated using remote sensing data only and Bayesian geostatistical models. Red indicates poorer areas in prediction maps, and higher error in uncertainty maps. 23

A. Input data

A.1. Geolocated survey data

The growing number of georeferenced household survey data from low and middle-income countries allows us to explore poverty metrics and comparisons between them while explicitly considering their geographic distribution. In Bangladesh, we utilised three geographically referenced datasets representing asset, consumption, and income-based measures of wellbeing (Figure S1).

A.1.1. Demographic and Health Survey Wealth Index

The Demographic and Health Surveys (DHS) were designed primarily to collect household data on marriage, fertility, family planning, reproductive and child health, and HIV/AIDS in almost all lower income countries[1]. Through the assembling of indicators correlated with a household's economic status (e.g. ownership of television, telephone, radio as well as variables describing type of floor and ceiling material and other facilities), a wealth index is calculated for each country at each time[2] based on the idea that the possession of assets and access to services and amenities are related to the relative economic position of the household in the country[3]. By its construction, the wealth index is a relative measure of wealth within each survey; however, a new methodology has been developed in order to make it comparable across countries and through time[4]. Moreover, recent adjustments have been made to the methods of constructing the wealth index to overcome criticism that the original score was not adequately capturing the differences between urban and rural poverty or identifying the poorest of the poor[3].

The wealth index is constructed using a principal component analysis (PCA), which includes a long list of assets owned by households as well as other indicators. (The complete list of indicators included in the PCAs for each survey, as well as PCA analysis and results can be found at http://dhsprogram.com/topics/wealth-index/Wealth-Index-Construction.cfm). The first factor from the PCA, capturing the largest percentage of the variance within the dataset, is derived adjusting for urban and rural strata[3,5]. In practice, a national index and two area-specific indexes representing urban and rural strata are individually constructed using sets of assets/services specific to each in order to better capture differences between urban and rural areas, and compare the wealth index between them[3]. Subsequently, applying regression techniques described in Rutstein[3] and Rutstein[6], the three indexes are combined into a single wealth distribution and a composite national index is derived. This method ensures comparability between urban and rural areas.

Here we used the 2011 Bangladesh DHS[7] (Figure S1*A*), a nationally representative survey based on a two-stage stratified sample of households, where 600 enumeration areas (EA or cluster) were first selected with probability proportional to the EA size, (207 clusters in urban areas and 393 in rural areas). This first stage of selection provided a listing of households for the second stage, where a systematic sample of 30 households on average was selected per cluster, to create statistically reliable estimates of key demographic and health variables[7,8]. In recent DHS surveys where HIV/AIDS data are not collected, geolocations for each cluster are available. The survey cluster coordinates represent an estimated centre of the cluster and are collected in the field through GPS receivers. To maintain respondents' confidentiality, GPS positions for all clusters are randomly displaced by a maximum of five kilometres for rural clusters and a maximum of two kilometres for urban clusters[9–11].

A.1.2. Progress out of Poverty Index

The Progress out of Poverty Index (PPI) (Figure S1*B*) was designed to be easily collected, simple and cost-effective to implement and verify[12], while applying a rigorous methodology through selecting assets based on their statistical relationship with poverty[12,13]. In the case of Bangladesh, an easy-to-use poverty scorecard[13] of 10 questions was created in March 2013, based on data from the Bangladesh 2010 Household Income and Expenditure Survey (HIES). The questions selected are aggregated into a score highly correlated with poverty status as measured by the HIES. The scores included in the scorecard are then translated into likelihoods that the household has per-capita expenditure above or below a given poverty line[13].

A nationally representative survey of all adults in Bangladesh was undertaken by InterMedia Financial Inclusion Insight Project (www.finclusion.org) in 2014 (wave 2), where 6,000 Bangladeshi individuals aged 15 and above were interviewed[14] and geolocations for each individual were included in the survey. In Bangladesh, InterMedia adopted a stratified sample strategy, whereby divisions and subdivisions were first identified and interviews within each subdivision were distributed in proportion to population size. In order to select the individuals to interview, households were first randomly selected using electoral rolls to randomly assign starting points in each selected subdivision. After having identified the starting point, subsequent households were selected using the right-hand rule, and the Kish Grid method was applied to select an individual respondent from each household[14,15].

A.1.3. Market research household surveys and income data

Two sequential large-scale market research household surveys were run by Telenor through its subsidiary, Grameenphone (GP), during 2 time periods between November and December 2013 (N=82,834, of which 55.3% GP subscribers) and February and March 2014 (N=87,509, of which 54.5% GP subscribers) (Figure S1*C*). The country was stratified in 226 sales territories by the phone company, and for every territory, an equal number of unions (in rural areas) and wards (in urban areas) were randomly selected. Four hundred households were surveyed in each territory, where a household was defined as a group of people sharing food from the same chula (fire/gas burner) or living under the same roof. Systematic sampling was then undertaken to select households by selecting every fourth household, starting from the selection of a random geographic point and direction within each ward or union. In the case of more than one household present in the complex or building, the fourth household was selected. In cases of non-response, the next household was then selected. Non-response rate was approximately 10% of households. Respondents within the household were selected via the Kish grid method[15] among those who were eligible. Eligibility was defined as individuals with their own phone, between 15 and 65 years of age. If a phone was shared between family members, usually the male head of household was interviewed. When the selected person was not home, the surveyors returned multiple times to try to reach the selected person. A very low non-response rate (less than 1-2%) was detected among respondents. The surveys were undertaken during working hours. To avoid that too many housewives were interviewed, given that men are more likely away for work, a ceiling on the number of housewives who could participate was also established. Sampling weights were applied to ensure national representativeness and correct for population sizes in urban and rural areas. Data quality control mechanisms were implemented and undertaken by the company; however, some sources of error were detected in matching household locations to phone number (approximately 20% of the cases).

A.2. Mobile phone call detail records (CDRs)

For the household income survey respondents described above, we collected 3-months of mobile phone metadata by subscriber consent. These metadata included call detail records (CDR) and top-up information, which were further processed into features. For each survey participant, 150 features from seven different feature families were constructed (Table S1). Household income was then linked to these metadata, resulting in three months of phone usage, matched with household income for each survey respondent. To preserve user anonymity, the local operator removes all personally identifying information from the data before analysis.

To be able to map poverty in other countries we focused on features that are easily reproducible, and easy to implement by local data warehouses. Most mobile operators generate similar features. CDR features range from metrics such as basic phone usage, top-up pattern, and social network to metrics of user mobility and handset usage. They include various parameters of the corresponding distributions such as weekly or monthly median, mean, and variance. In addition, we received pre-aggregated datasets of tower-level activity from 48,190,926 subscriber SIMs over a 4-month period. This includes monthly number of subscribers per home cell, where home cell corresponds to most frequent tower. These per-user features are not directly used, but further aggregated to the Voronoi polygons, and the aggregate features are used in covariate selection, model fitting, and prediction.

At the time of data acquisition, the mobile phone operator had an approximate 42% market share, and was the largest provider of mobile telecommunication services in Bangladesh. Multi-SIM activity is common in Bangladesh, but we believe that this should not create a systematic bias in poverty estimates because the geographic coverage of the operator is so extensive. In order to comply with national laws and regulations of Bangladesh, and the privacy policy of the Telenor group, the following measures were implemented in order to preserve the privacy rights of Grameenphone customers:

> 1) All customers are de-identified and only Telenor/Grameenphone employees have had access to any detailed CDR-/top-up data;
> 2) The processing of detailed CDR/top-up data resulted in aggregations of the data on a tower-level granularity; the tower-level aggregation makes re-identification impossible.

Hence, the resulting aggregated dataset is truly anonymous and involves no personal data.

Compared with other countries of comparable income levels, Bangladesh has a high mobile phone penetration, which includes rural areas. Fifty percent of the population above the age of fifteen has a mobile subscription[16]. The proportion of households with at least one mobile phone is increasing rapidly; between 2011 and 2014, household ownership across the whole of Bangladesh rose from 78% to 89%, with much of that growth concentrated among rural households[17]. The CDR data used in this study are available upon request for the replication of results only by contacting the corresponding author.

A.3. Remote Sensing-GIS covariates

Ancillary data layers used as remote sensing-GIS (hereafter RS) covariates were identified, assembled, and processed for the whole of Bangladesh at a 1-km spatial resolution. These data are described in Table S1 and include 25 raster and vector datasets obtained from existing sources or produced ad hoc for this study to include environmental and physical metrics likely to be associated with human welfare[18–22]. These data differed in spatial and temporal resolution, type, accuracy, and coverage. In order to align all data for model fitting and prediction, the following steps were taken:

1) Bangladesh was rasterized at a resolution of 30-arcsec (0.00833333 degree, corresponding to approximately 1-km at the equator);
2) Vector datasets were rasterized at a resolution of 30-arcsec;
3) When necessary, raster datasets were resampled to a resolution of 30-arcsec using an interpolation technique appropriate for the resolution and type of the original dataset;
4) All datasets were spatially aligned to make every pixel representing the same location coincident and match the rasterized study area.

Furthermore, for ad hoc datasets such as distance to roads and waterways, we used a customized Azimuthal Equidistant projection centred in the middle of the study area and clipped to a buffer extending 100 metres beyond its boundary to project the input data. This buffered area was rasterized to a resolution of approximately 927 metres, corresponding to 30-arcsec at the centre of the study area where distortion is smallest. Euclidean distance was calculated for each distance-to covariate within the customized projection. The resultant layers were then projected back to GCS WGS84, and made coincident with the rasterized study area. All datasets representing categorical variables (e.g. protected areas, global urban extent, etc.) were projected, rasterized, and/or resampled to 1-km resolution, spatially aligned to the rasterized study area, and converted into binary covariates, representing the presence or absence of a given feature. This resulted in twenty-five 1-km raster datasets, which were used to extract the mean, mode, or sum of each covariate for each Voronoi polygon, dependent on the type of dataset. These values were used for covariate selection, model fitting, and prediction.

A.3.1 GPS data displacement

In addition to the aforementioned processing, additional steps were undertaken to appropriately account for the displacement inherent in DHS data. When these data are collected, the latitude and longitude of the centre of each DHS cluster (representing numerous households) is collected in the field with a GPS receiver. To maintain respondents' confidentiality, GPS latitude/longitude positions for all DHS clusters are randomly displaced by a maximum of five kilometres for rural clusters and two kilometres for urban clusters. The displacement is restricted so the points stay within the country, within the DHS survey region, and within the second administrative level[9–11].

In order to account for the displacement in our analyses, we created buffers around each cluster centroid of 2 km and 5 km for urban and rural clusters, respectively, and subsequently extracted the RS covariate data for each buffer zone. For continuous covariates, the minimum, maximum, and mean values were calculated and extracted. For categorical covariates, the modal value was calculated and extracted.

B. Statistical analyses and prediction mapping

B.1. Covariate selection via generalized linear models

Stratifying models into urban and rural components produced the best fit models as measured by AIC. Top-up data produced the most important CDR feature family for all poverty measures and models. Within this feature family, significant covariates included recharge amounts and frequencies per tower, spending speeds and time between refills, and fractions of the lowest and highest available top-up amounts. Advanced phone usage was also an important CDR feature family, especially for PPI and income models. Sum, revenue, count, and volume of ingoing and outgoing multimedia messaging, Internet usage, and videos were prominent. Basic phone usage covariates measuring incoming and outgoing text counts were important for every model save for rural WI models. Mobility covariates including number and entropy of places and radius of gyration were also significant features in all three strata and poverty measures, as were social network features such as number and entropy of contacts.

Nighttime lights and covariates representing access - especially transport time to closest urban settlement and distance to roads - were the most important RS covariates for all three poverty measures and strata. Vegetation productivity, as measured by the Enhanced Vegetation Index (EVI), and elevation were also prominent RS features in all three strata, whereas climate variables featured prominently in rural models.

B.2. Prediction mapping via Bayesian geostatistical models

Using the models selected and described in Tables S2*A-C*, we employed hierarchical Bayesian geostatistical models (BGMs) for prediction as described in our manuscript. All prediction maps not highlighted in our manuscript can be found in Figures S2-S6. Model performance was based on out-of-sample validation statistics calculated on a 20% test subset of poverty data input points (see Table 1 in manuscript). The performance of models built with CDR-only or RS-only data varied based on poverty measure and strata. RS-only models were more successful at predicting the WI for all three strata (r^2= 0.74, 0.71, 0.72 for national, urban, and rural models), as compared to CDR-only models. However, the CDR-only models performed nearly as well (r^2= 0.64, 0.70, 0.50 for national, urban, and rural models), and all urban WI models including CDRs outperformed national level models. The urban CDR-RS model exhibits the highest explained variance for any model (r^2=0.78), and the urban CDR-only model outperforms the national CDR-only model (r^2=0.70 versus r^2=0.64, respectively). For PPI and income measures of poverty, CDR data produced the best models in urban areas, whereas RS data produced the best models in rural areas. This highlights the compatibility of these two datasets for predicting different measures of poverty at different scales, as the best estimates and lowest error corresponded to the data with fine-scale spatial heterogeneity (CDRs in urban areas; RS data in rural areas). To that end, national poverty models generally performed best when utilising both CDRs and RS data.

To compare full model performance against a spatial interpolation model, we modelled the training data for all three poverty indicators using only the spatial random effect in the INLA model (see section 2.5 in manuscript). These results are shown in Table S3. We compared out-of-sample r^2 and RMSE values against results from the full models (see Table 1 in manuscript). The results show a spatial pattern in the WI data as the model built with only a spatial random effect yields an r^2=0.49, RMSE=0.578. When compared to the full model, the addition of covariate data increases the r^2 to 0.76, and the RMSE decreases to 0.394. Similarly for income, the data do show a slight spatial pattern, but the addition of covariate data to the model increases the predictive power and

decreases the error. For the PPI, the covariates do not show a strong influence in the modelling results, and the model was driven by the spatial process, which suggests there's an underlying spatial covariate that we're not capturing in the model that could explain the data.

Model fit based on the spatial effect can also be considered using DIC, a hierarchical modelling generalization of the AIC and BIC, which can be useful in Bayesian modelling comparison. The BIC allows for comparing models using criterion based on the trade-off between the fit of the data to the model and the corresponding complexity of the model. Models with smaller DIC values are preferred over models with larger DIC values as the measure favours better fit and fewer parameters[23]. These results are shown in Table S4. For nearly every model with CDR data, DIC is greatly improved by accounting for the spatial covariance in the data structure. However, the income models see slight or no improvement from including the random spatial effect, likely due to the fact that they include and are thus penalised for many covariates.

References

1. Rutstein, S. & Rojas, G. *Guide to DHS Statistics*. 168 (ORC Macro, Demographic and Health Surveys, 2006).
2. Rutstein, S. & Johnson, K. *The DHS Wealth Index*. (ORC Macro, Demographic and Health Surveys, 2004).
3. Rutstein, S. *The DHS Wealth Index: Approaches for Rural and Urban Areas.* (United States Agency for International Development, 2008).
4. Rutstein, S. & Staveteig, S. *Making the Demographic and Health Surveys Wealth Index Comparable*. (ICF International).
5. Rogers, D., Wint, G., Alexander, N., Pozzi, F. & Robinson, T. *Wealth Index Mapping in the Horn of Africa*. (Food and Agriculture Organization of the United Nations, 2011).
6. Rutstein, S. *Steps to constructing the new DHS Wealth Index*.
7. National Institute of Population Research and Training, Mitra and Associates & ICF International. *Bangladesh Demographic and Health Survey 2011*. (2013).
8. ICF International. *Demographic and Health Survey Sampling Household Listing Manual*. (ICF International, 2012).

9. Burgert, C., Colston, J., Thea Roy, T. & Zachary, B. *Geographic displacement procedure and georeferenced data release policy for the Demographic and Health Surveys*. (ICF International, 2013).
10. Perez-Heydrick, C., Warren, J., Burgert, C. & Emch, M. *Guidelines on the Use of DHS GPS Data*. (ICF International, 2013).
11. DHS Spatial Interpolation Working Group. *Spatial Interpolation with Demographic and Health Survey Data: Key Considerations*. (ICF International, 2014).
12. Desiere, S., Vellema, W. & D'Haese, M. A validity assessment of the Progress out of Poverty Index (PPI)™. *Eval. Program Plann.* **49,** 10–18 (2015).
13. Schreiner, M. *A Simple Poverty Scorecard for Bangladesh*. (2013).
14. Intermedia. *Intermedia Financial Inclusion Insight Project Bangladesh - Steps Toward Financial Inclusion 2014*. (2014).
15. Kish, L. A Procedure for Objective Respondent Selection within the Household. *J. Am. Stat. Assoc.* **44,** 380–387 (1949).
16. Lucini, B. A. & Hatt, T. *Country overview: Bangladesh*. (GSMA Intelligence, 2014).
17. National Institute of Population Research and Training, Mitra and Associates & The DHS Program. *Bangladesh Demographic and Health Survey 2014: Key Indicators*. (NIPORT, Mitra and Associates, and ICF International, 2015).
18. Pozzi, F., Robinson, T. & Nelson, A. *Accessibility Mapping and Rural Poverty in the Horn of Africa*. (Food and Agriculture Organization of the United Nations, 2009).
19. Rogers, D., Emwanu, T. & Robinson, T. *Poverty Mapping in Uganda: An Analysis Using Remotely Sensed and Other Environmental Data*. (Food and Agriculture Organization of the United Nations, 2006).
20. Noor, A. M., Alegana, V. A., Gething, P. W., Tatem, A. J. & Snow, R. W. Using remotely sensed night-time light as a proxy for poverty in Africa. *Popul. Health Metr.* **6,** 5 (2008).

21. Ghosh, T., Anderson, S. J., Elvidge, C. D. & Sutton, P. C. Using Nighttime Satellite Imagery as a Proxy Measure of Human Well-Being. *Sustainability* **5,** 4988–5019 (2013).
22. Watmough, G. R., Atkinson, P. M., Saikia, A. & Hutton, C. W. Understanding the Evidence Base for Poverty–Environment Relationships using Remotely Sensed Satellite Data: An Example from Assam, India. *World Dev.* **78,** 188–203 (2016).
23. Spiegelhalter, D. J., Best, N. G., Carlin, B. P. & Van Der Linde, A. Bayesian measures of model complexity and fit. *J. R. Stat. Soc. Ser. B Stat. Methodol.* **64,** 583–639 (2002).

SI Tables and Figures

Table S1. Summary information for remote sensing-GIS and mobile phone call detail record datasets used for covariate selection and Bayesian geostatistical poverty mapping.

Category	Description	Source	Resolution (Degrees)	Year
		**RS-GIS**		
Accessibility	Accessibility to populated places with more than 50k people	European Commission Joint Research Centre (http://forobs.jrc.ec.europa.eu/products/gam/)	0.0083333	2000
Population	Population count [per pixel]	WorldPop (http://www.worldpop.org.uk/)	0.0008333	2010
Population	Population count [per pixel]	CIESIN - Global Rural Urban Mapping Project (http://sedac.ciesin.columbia.edu/data/collection/grump-v1/sets/browse)	0.0083333	2000
Population	Population density [per sqkm]	CIESIN - Global Rural Urban Mapping Project (http://sedac.ciesin.columbia.edu/data/collection/grump-v1/sets/browse)	0.0083333	2000
Climate	Mean Aridity Index	CGIAR-CSI (http://www.cgiar-csi.org/data)	0.0083333	1950-2000
Climate	Average annual Potential Evapotranspiration [mm]	CGIAR-CSI (http://www.cgiar-csi.org/data)	0.0083333	1950-2000
Night-time lights	VIIRS night-time lights [W cm-2 sr-1]	NOAA VIIRS (http://ngdc.noaa.gov/eog/viirs.html)	0.0041667	2014
Elevation	Elevation [meter]	CGIAR-CSI (http://srtm.csi.cgiar.org/)	0.0083316	2008
Vegetation	Vegetation	MODIS MOD13A1 [Enhanced vegetation index]	0.0041667	2010-2014
Distance	Distance to roads [meter]	Input data from OSM (http://extract.bbbike.org/)	0.0083333	2014
Distance	Distance to waterways [meter]	Input data from OSM (http://extract.bbbike.org/)	0.0083333	2014
Urban/Rural	Urban/Rural	MODIS-based Global Urban extent	0.0041670	2000-2001
Urban/Rural	Urban/Rural	CIESIN - Global Rural Urban Mapping Project (http://sedac.ciesin.columbia.edu/data/collection/grump-v1/sets/browse)	0.0083333	2000
Urban/Rural	Global Human Settlement Layer	Global Land Cover Facility (www.landcover.org)	0.002818	2014
Protected Area	Protected areas	WDPA (http://www.protectedplanet.net/)	Vector	2012
Land Cover	Land cover	ESA GlobCover Project (http://due.esrin.esa.int/page_globcover.php)	0.0027777	2009
Land Cover	Land cover	IGBP MODIS MCD12Q1 (https://lpdaac.usgs.gov/dataset_discovery/modis/modis_products_table/mcd12q1)	0.0041670	2012
Land Cover	Land cover	ONRL DAAC Synergetic Land Cover Product (SYNMAP) (http://webmap.ornl.gov/wcsdown/wcsdown.jsp?dg_id=10024_1)	0.0083333	2000-2001

Demographic	Pregnancies	WorldPop (http://www.worldpop.org.uk/)	0.0008333	2012
Demographic	Births	WorldPop (http://www.worldpop.org.uk/)	0.0008333	2012
Ethnicity	Georeferenced ethnic groups	ETH Zurich (http://www.icr.ethz.ch/data/geoepr)	Vector	2014
Climate	Mean annual precipitation	WorldClim (http://www.worldclim.org/download)	0.0083333	1950-2000
Climate	Mean annual temperature	WorldClim (http://www.worldclim.org/download)	0.0083333	1950-2000
**Call Detail Records**				
Basic phone usage	Outgoing/incoming voice duration, SMS count, etc.	Telenor/Grameenphone	NA	2013-2014
Top-up transactions	Spending speed, recharge amount per transaction, fraction of lowest/highest recharge amount, coefficient of variation recharge amount, etc.	Telenor/Grameenphone	NA	2013-2014
Location/mobility	Home district/tower, radius of gyration, entropy of places, number of places, etc.	Telenor/Grameenphone	NA	2013-2014
Social Network	Interaction per contact, degree, entropy of contacts, etc.	Telenor/Grameenphone	NA	2013-2014
Handset type	Brand, manufacturer, camera enabled, smart/feature/basic phone, etc.	Telenor/Grameenphone	NA	2013-2014
Revenue	Charge of outgoing/incoming SMS, MMS, voice, video, value added services, roaming, internet, etc.	Telenor/Grameenphone	NA	2013-2014
Advanced phone usage	Internet volume/count, MMS count, video count/duration, value added services duration/count, etc.	Telenor/Grameenphone	NA	2013-2014

Table S2*A*. Wealth Index models for RS-only, CDR-only, and CDR+RS data: national, urban, and rural strata.

**AIC**	NATIONAL	URBAN	RURAL
RS-only	690.61	333.44	161.42
CDR-only	907.78	373.97	164.14
CDR-RS	651.76	318.12	115.52
**MODEL**			
RS-only	1 + transport time to closest urban settlement + nighttime lights + EVI + elevation	1 + distance to roads + distance to waterways + nighttime lights + elevation	1 + transport time to closest urban settlement + annual temperature + annual precipitation + distance to roads + distance to waterways + nighttime lights
CDR-only	1 + recharge average per tower + percent nocturnal calls + number of places + entropy of contacts + outgoing internet sessions + sum outgoing internet sessions + incoming voice duration + count incoming content management system + count sum incoming content management system + volume of incoming multimedia messages + recharge amount per transaction + count incoming multimedia messages + count incoming texts + weekly recharge amount	1 + recharge average per tower + number of places + entropy of contacts + spending speed + average outgoing text count + sum count incoming content management system + weekly recharge amount	1 + recharge average per tower + percent nocturnal calls + entropy of places + radius of gyration + interactions per contact + recharge amount (CV) + number of retailers visited weekly (CV) + sum incoming video duration + count incoming multimedia messages + weekly recharge frequency (CV) + sum incoming video count + recharge amount per transaction (CV)
CDR-RS	1 + transport time to closest urban settlement + nighttime lights + EVI + elevation + recharge average per tower + percent nocturnal calls + outgoing internet sessions + count incoming content management system + recharge amount per transaction + count incoming texts + weekly recharge amount	1 + distance to roads + distance to waterways + nighttime lights + elevation + recharge average per tower + spending speed + average outgoing text count + weekly recharge amount	1 + transport time to closest urban settlement + annual temperature + distance to roads + distance to waterways + nighttime lights + recharge average per tower + percent nocturnal calls + entropy of places + radius of gyration + interactions per contact + recharge amount (CV) + number of retailers visited weekly (CV) + sum incoming video duration + weekly recharge frequency (CV) + sum incoming video count + recharge amount per transaction (CV)

*CV=coefficient of variation

Table S2*B*. Progress out of Poverty Index models for RS-only, CDR-only, and CDR+RS data: national, urban, and rural strata.

**AIC**	NATIONAL	URBAN	RURAL
RS-only	41676	14099	27465
CDR-only	41562	14044	27421
CDR-RS	41502	14043	27382
**MODEL**			
RS-only	1 + annual precipitation + annual temperature + transport time to closest urban settlement + distance to roads + EVI + nighttime lights	1 + annual precipitation + annual temperature + transport time to closest urban settlement + elevation	1 + annual precipitation + annual temperature + transport time to closest urban settlement + distance to water + EVI + nighttime lights
CDR-only	1 + subscribers per tower + recharge average per tower + entropy of places + entropy of contacts + average outgoing text count + sum outgoing multimedia messages + fraction of 10 Thaka top-ups (min amount) + outgoing internet sessions + sum outgoing internet sessions + sum count incoming content management system + number of retailers visited weekly (CV) + sum revenue outgoing multimedia messages + spending speed variance + count incoming multimedia messages + sum count incoming multimedia messages + count incoming texts + sum count incoming texts + weekly recharge frequency (CV) + median time between refills + incoming video count + outgoing internet volume + time variable (CV)	1 + subscribers per tower + recharge average per tower + sum outgoing multimedia messages + count outgoing internet sessions + sum count outgoing internet sessions + number of retailers visited weekly (CV) + volume of incoming multimedia messages + outgoing text charges + sum revenue outgoing multimedia messages + incoming video duration + sum incoming video duration + spending speed variance + sum count incoming multimedia messages + weekly recharge amount + incoming video count + sum incoming video count + number of retailers visited weekly	1 + subscribers per tower + recharge average per tower + number of places + entropy of places + sum duration outgoing value added services + count outgoing texts + sum count outgoing texts + sum volume of outgoing multimedia messaging + fraction of 300 Thaka top-ups + count outgoing internet sessions + sum count outgoing internet sessions + incoming voice duration + number of retailers visited weekly (CV) + volume of incoming multimedia messages + outgoing text charges + sum outgoing text charges + sum revenue outgoing multimedia messages + spending speed variance + count incoming texts + sum count incoming texts + weekly recharge frequency (CV) + median time between refills + outgoing internet volume + recharge amount per transaction (CV) + time variable (CV)
CDR-RS	1 + annual precipitation + annual temperature + transport time to closest urban settlement + distance to road + elevation + subscribers per tower + recharge average per tower + entropy of places + entropy of contacts + average outgoing text count + sum outgoing multimedia messages + outgoing internet sessions + sum outgoing internet sessions + number of retailers visited weekly (CV) + sum revenue outgoing multimedia messages + spending speed variance + count incoming multimedia messages + sum count incoming multimedia messages + count incoming texts + sum count incoming texts + weekly recharge frequency (CV) + median time between refills + incoming video count + outgoing internet volume + time variable (CV)	1 + annual precipitation + annual temperature + EVI + elevation + subscribers per tower + recharge average per tower + sum outgoing multimedia messages + number of retailers visited weekly (CV) + volume of incoming multimedia messages + sum volume of incoming multimedia messages + incoming video duration + sum incoming video duration + sum revenue outgoing multimedia messages + spending speed variance + sum count incoming multimedia messages + weekly recharge amount + incoming video count + sum incoming video count + number of retailers visited weekly	1 + annual precipitation + annual temperature + transport time to closest urban settlement + distance to water + EVI + nighttime lights + subscribers per tower + recharge average per tower + outgoing multimedia messages + fraction of 300 Thaka top-ups + number of retailers visited weekly (CV) + volume of incoming multimedia messages + outgoing text charges + sum revenue outgoing multimedia messages + spending speed variance + median time between refills + time variable (CV)

*CV=coefficient of variation

Table S2*C*. Income models for RS-only, CDR-only, and CDR+RS data: national, urban, and rural strata.

**AIC**	NATIONAL	URBAN	RURAL
RS-only	83194	17295	64569
CDR-only	83109	17180	64413
CDR+RS	82895	17129	64330
**MODEL**			
RS-only	1 + nighttime lights + transport time to closest urban settlement + annual temperature + EVI + distance to raod + distance to water + annual precipitation + elevation	1 + nighttime lights + transport time to closest urban settlement + distance to roads	1 + nighttime lights + annual temperature + distance to roads + annual precipitation + elevation
CDR-only	1 + percent nocturnal calls + number of places + entropy of places + entropy of contacts + radius of gyration + interactions per contact + spending speed + outgoing video duration + sum outgoing video duration + average outgoing text count + sum outgoing text count + fraction of 300 Thaka top-ups + fraction of 10 Thaka top-ups + recharge amount (CV) + number of retailers visited weekly (CV) + recharge amount per transaction + spending speed variance + sum spending speed variance + sum count incoming texts + handset weight + outgoing voice duration + sum outgoing voice duration + sum outgoing internet volume + time variable (CV) + fraction of 200 Thaka top-ups	1 + percent nocturnal calls + number of places + entropy of places + radius of gyration + spending speed + outgoing video duration + sum outgoing video duration + average outgoing text count + sum outgoing text count + fraction of 300 Thaka top-ups + number of retailers visited weekly (CV) + volume of incoming multimedia messages + sum volume of incoming multimedia messages + recharge amount per transaction + count incoming multimedia messages + sum count incoming multimedia messages + sum outgoing voice duration + outgoing internet volume + sum outgoing internet volume + recharge amount per transaction (CV) + time variable (CV)	1 +number of places + entropy of places + entropy of contacts + spending speed + duration outgoing value added services + sum duration outgoing value added services + sum outgoing video duration + handset weight + software OS version + fraction of 300 Thaka top-ups + fraction of 10 Thaka top-ups + coefficient of variation: recharge amount + number of retailers visited weekly (CV) + volume of incoming multimedia messages + sum volume of incoming multimedia messages + spending speed variance + sum spending speed variance + sum count incoming multimedia messages + count incoming texts + sum count incoming texts + weekly recharge amount + outgoing voice duration + sum outgoing voice duration + outgoing internet volume + time variable (CV)
CDR-RS	1 + nighttime lights + transport time to closest urban settlement + EVI + distance to road + percent nocturnal calls + number of places + entropy of places + entropy of contacts + radius of gyration + spending speed + outgoing video duration + sum outgoing video duration + average outgoing text count + sum outgoing text count + recharge amount (CV) + number of retailers visited weekly (CV) + recharge amount per transaction + spending speed variance + sum spending speed variance + sum count incoming texts + handset weight + outgoing voice duration + sum outgoing voice duration + sum outgoing internet volume + time variable (CV) + fraction of 200 Thaka top-ups	1 + nighttime lights + transport time to closest urban settlement + annual temperature + distance to roads + annual temperature + percent nocturnal calls + number of places + radius of gyration + spending speed + outgoing video duration + sum outgoing video duration + average outgoing text count + sum outgoing text count + fraction of 300 Thaka top-ups + number of retailers visited weekly (CV) + volume of incoming multimedia messages + recharge amount per transaction + count incoming multimedia messages + sum count incoming multimedia messages + outgoing voice duration + outgoing internet volume + sum outgoing internet volume + recharge amount per transaction (CV) + time variable (CV)	1 + nighttime lights + annual precipitation + number of places + entropy of places + entropy of contacts + spending speed + sum outgoing video duration + handset weight + software OS version + fraction of 300 Thaka top-ups + fraction of 10 Thaka top-ups + coefficient of variation: recharge amount + number of retailers visited weekly (CV) + weekly recharge amount + outgoing voice duration + sum outgoing voice duration + outgoing internet volume + time variable (CV)

*CV=coefficient of variation

Table S3. Comparison of r^2 and RMSE for INLA models run with only a structured spatial random effect (Spatial interpolation) and the full model (Spatial model + covariates).

Poverty Metric	Spatial interpolation	Spatial model + covariates (from Table 1)
	R^2, RMSE	R^2, RMSE
DHS WI	0.49, 0.578	0.76, 0.394
PPI	0.31, 58.727	0.32, 57.439
Income	0.10, 123.963	0.27, 105.465

Table S4. Comparison of deviance information criterion (DIC) model fit for Bayesian geostatistical models run with a structured spatial random effect (Spatial model) and without (Non-spatial model).

Poverty Metric	Model	Spatial model DIC	Non-spatial model DIC
**WHOLE COUNTRY**			
DHS WI	CDR - RS	463.7	574.6
	CDR	272.5	862.2
	RS	465.7	581.5
PPI	CDR - RS	1361.3	1439.8
	CDR	1349.7	1473.1
	RS	1358.6	1421.4
Income	CDR - RS	66142.5	66143.0
	CDR	66314.6	66314.3
	RS	66482.6	66480.4
**URBAN**			
DHS WI	CDR - RS	449.4	576.0
	CDR	239.2	873.2
	RS	454.1	582.0
PPI	CDR - RS	1371.6	1432.3
	CDR	1365.7	1470.1
	RS	1358.3	1417.7
Income	CDR - RS	66180.6	66179.8
	CDR	66363.3	66365.7
	RS	66693.0	66690.3
**RURAL**			
DHS WI	CDR - RS	458.0	574.5
	CDR	63.1	873.5
	RS	451.5	595.5
PPI	CDR - RS	1376.9	1444.6
	CDR	1342.9	1475.9
	RS	1357.7	1419.4
Income	CDR - RS	66262.5	66260.6
	CDR	66395.0	66392.1
	RS	65548.9	66503.8

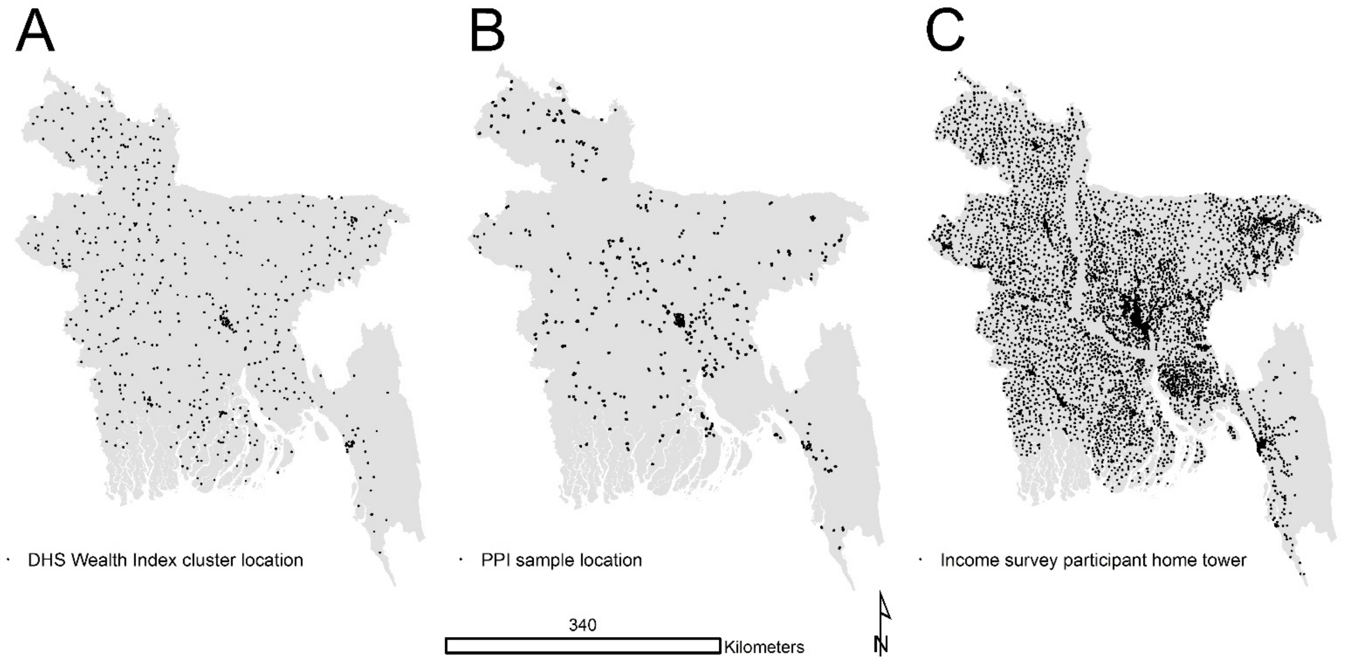

Figure S1. Survey sample locations for DHS wealth index (A), Progress out of Poverty Index (B), and income survey respondents (C).

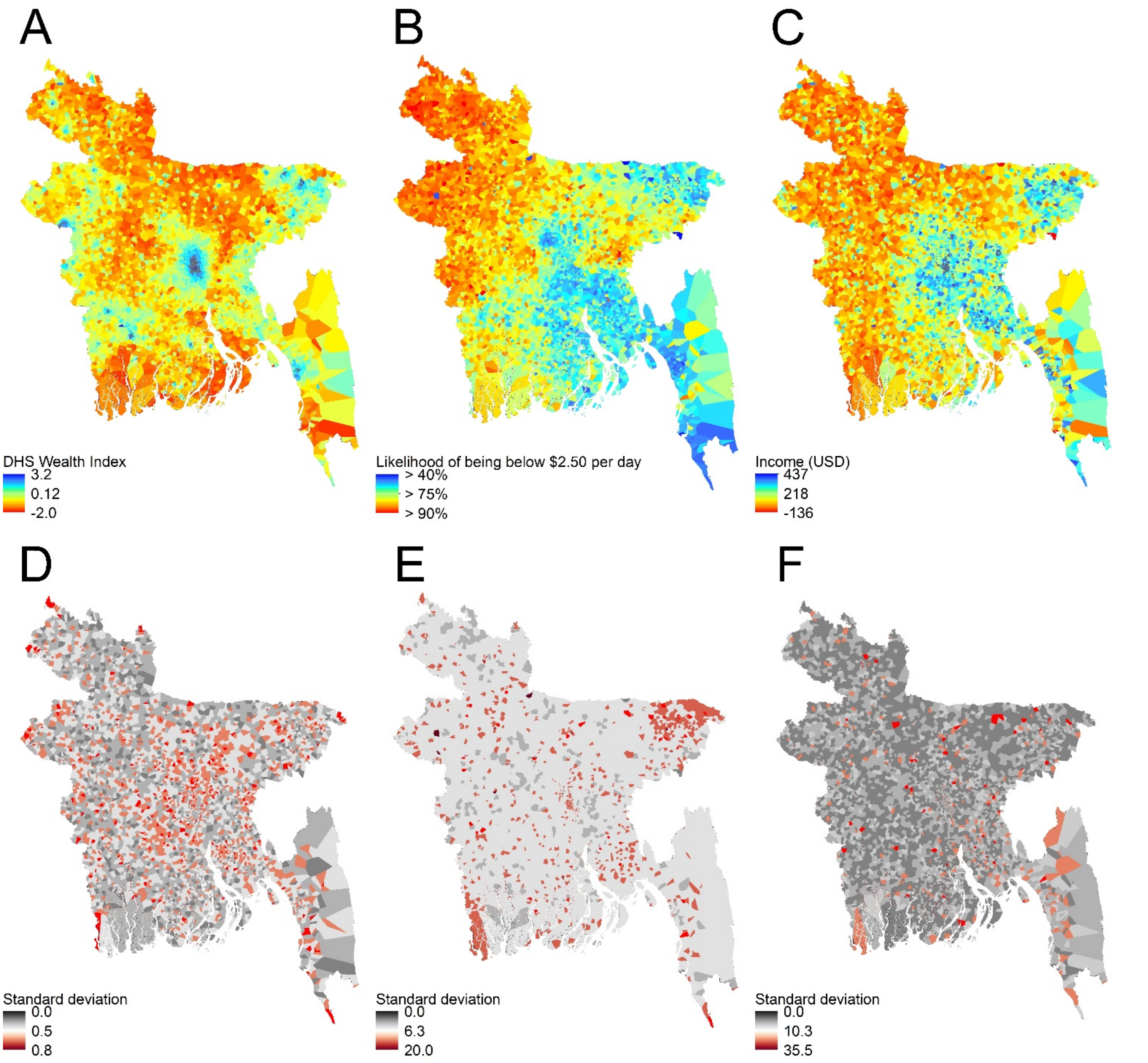

Figure S2. National level prediction maps for mean wealth index (A) with uncertainty (D); mean probability of households being below $2.50/day (B) with uncertainty (E); and mean USD income (C) with uncertainty (F). Maps were generated using call detail record features only and Bayesian geostatistical models. Red indicates poorer areas in prediction maps, and higher error in uncertainty maps.

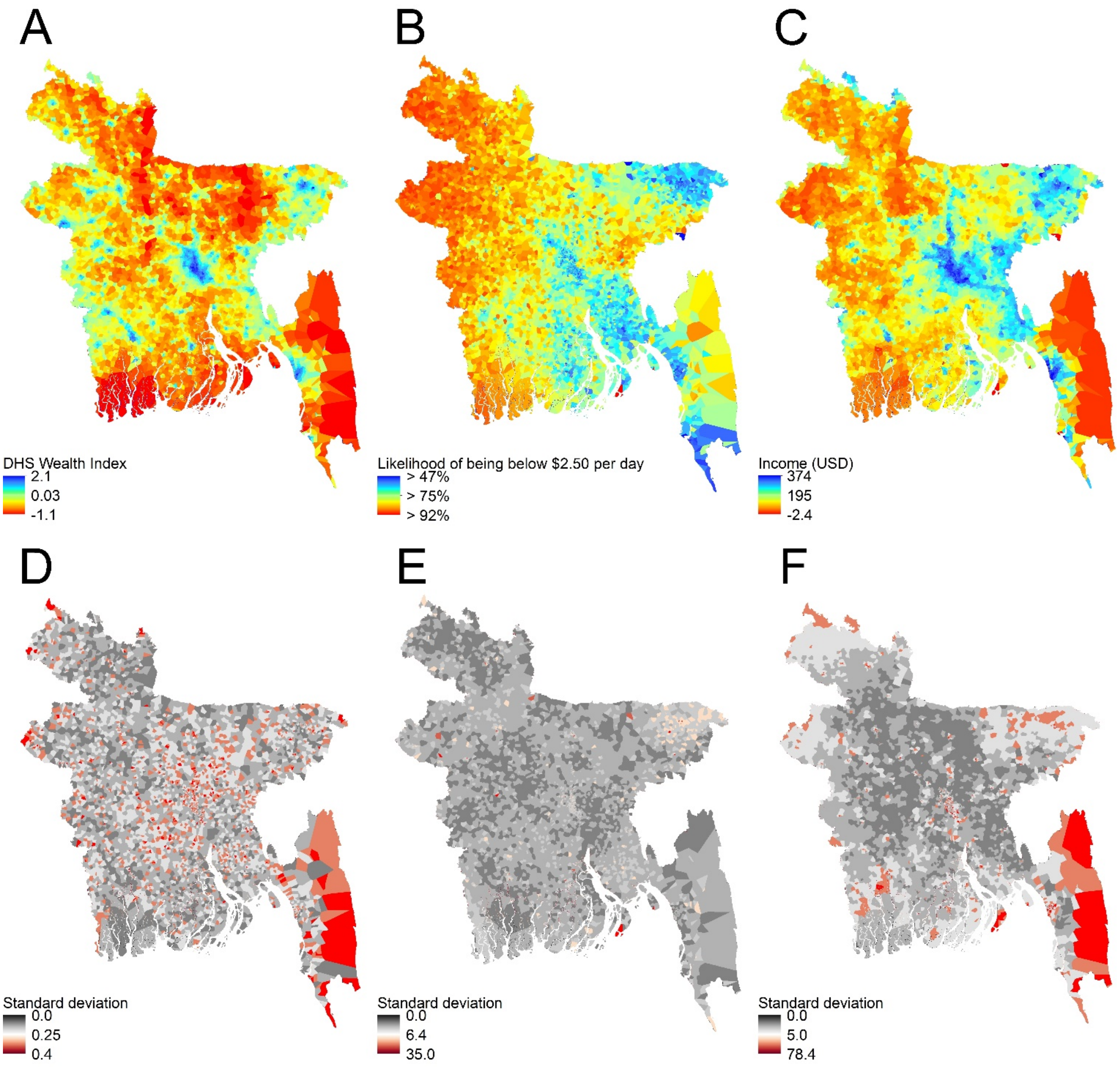

Figure S3. National level prediction maps for mean wealth index (A) with uncertainty (D); mean probability of households being below $2.50/day (B) with uncertainty (E); and mean USD income (C) with uncertainty (F). Wealth index and income maps were generated using remote sensing data only; PPI maps were generated using call detail record features and remote sensing data. All maps were generated using Bayesian geostatistical models. Red indicates poorer areas in prediction maps, and higher error in uncertainty maps.

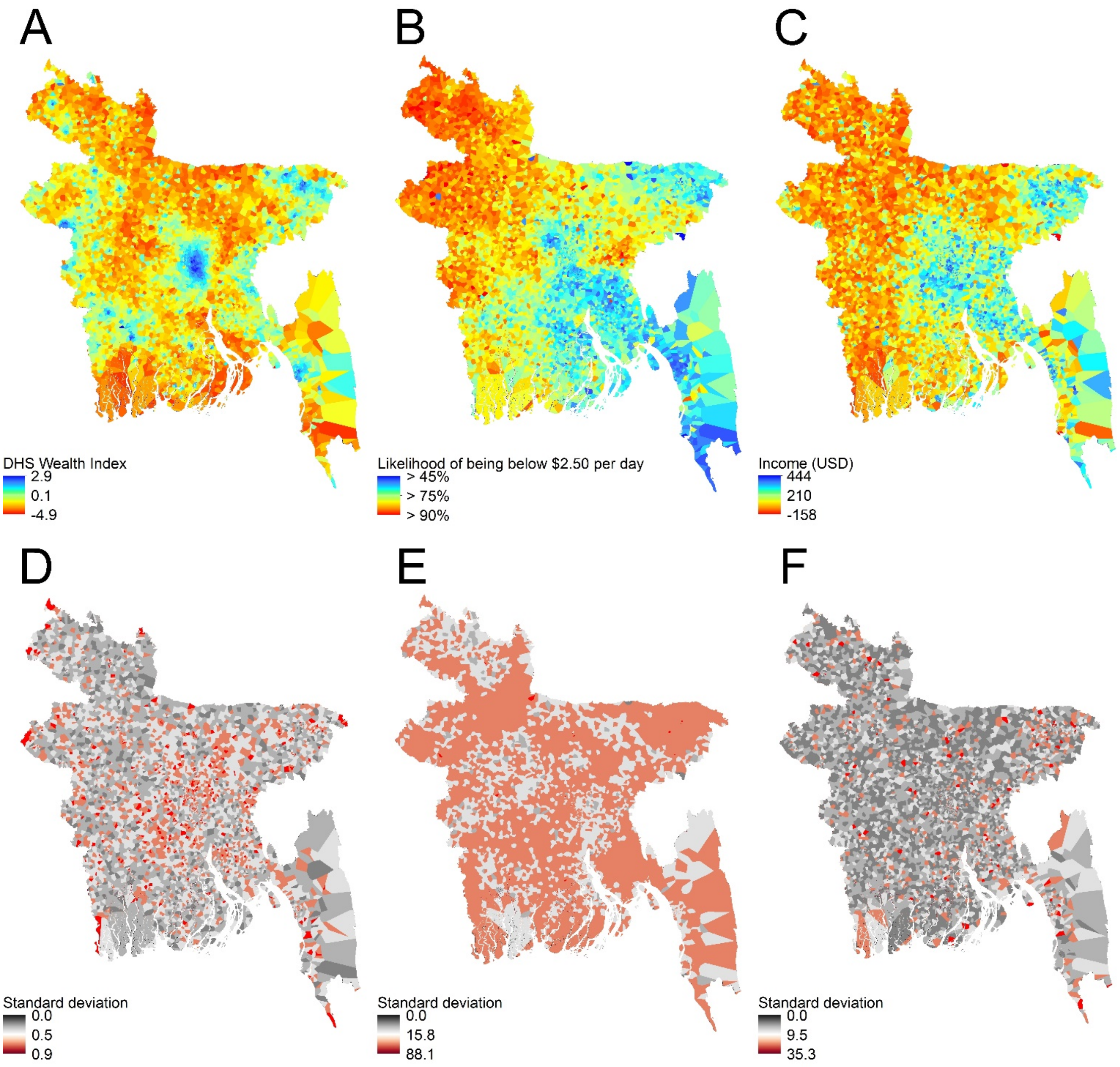

Figure S4. Stratified urban/rural prediction maps for mean wealth index (A) with uncertainty (D); mean probability of households being below $2.50/day (B) with uncertainty (E); and mean USD income (C) with uncertainty (F). Maps were generated using call detail record features only and Bayesian geostatistical models. Red indicates poorer areas in prediction maps, and higher error in uncertainty maps.

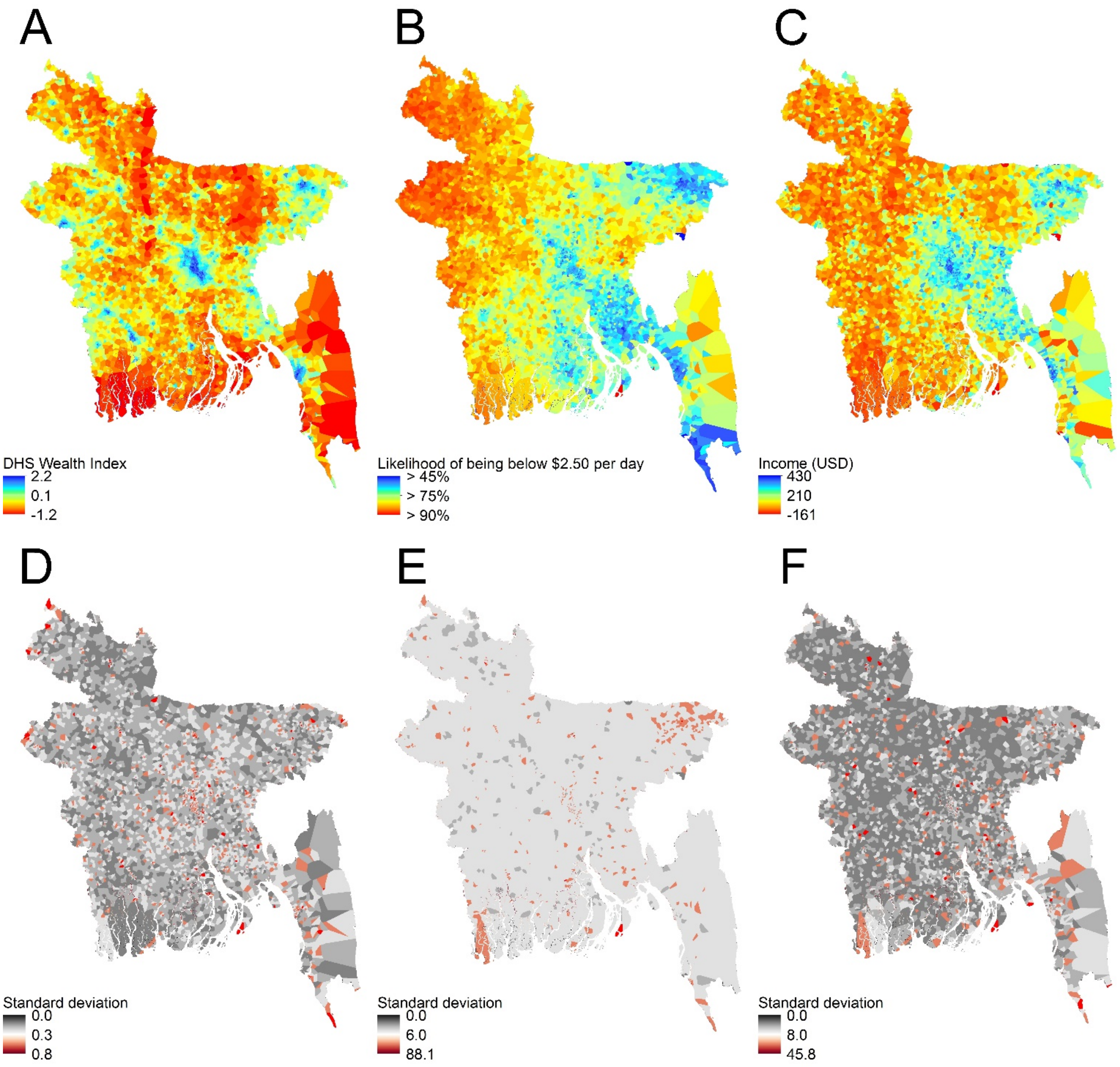

Figure S5. Stratified urban/rural prediction maps for mean wealth index (A) with uncertainty (D); mean probability of households being below $2.50/day (B) with uncertainty (E); and mean USD income (C) with uncertainty (F). Maps were generated using call detail record features, remote sensing data, and Bayesian geostatistical models. Red indicates poorer areas in prediction maps, and higher error in uncertainty maps.

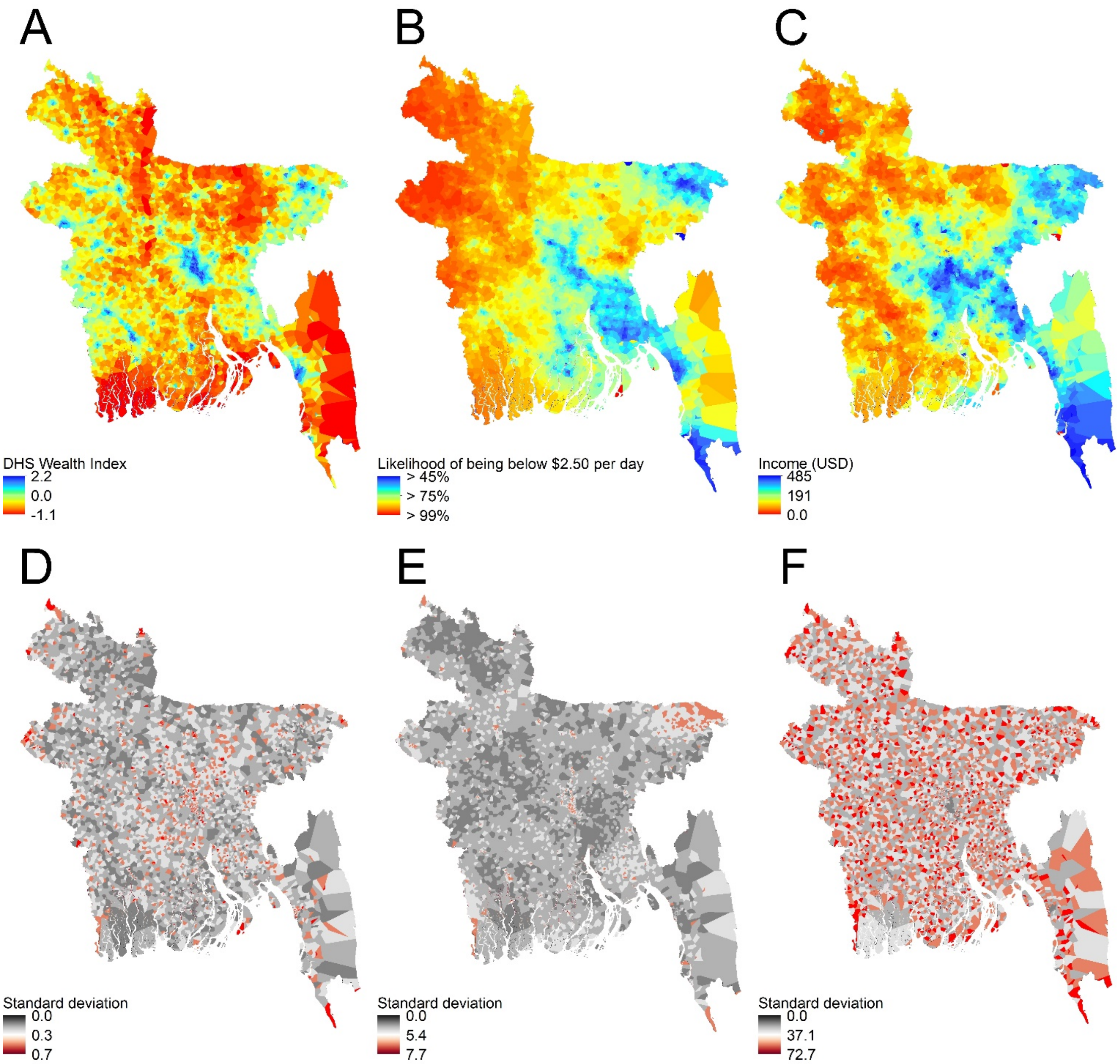

Figure S6. Stratified urban/rural prediction maps for mean wealth index (A) with uncertainty (D); mean probability of households being below $2.50/day (B) with uncertainty (E); and mean USD income (C) with uncertainty (F). Maps were generated using remote sensing data only and Bayesian geostatistical models. Red indicates poorer areas in prediction maps, and higher error in uncertainty maps.
